# Prefracture functional level evaluated by the New Mobility Score predicts in-hospital outcome after hip fracture surgery

**DOI:** 10.3109/17453674.2010.487240

**Published:** 2010-05-21

**Authors:** Morten T Kristensen, Nicolai B Foss, Charlotte Ekdahl, Henrik Kehlet

**Affiliations:** ^1^Division of Physical Therapy, Health Sciences Center, Lund UniversitySweden; ^2^Departments of Physical Therapy; ^3^Orthopedic Surgery; ^4^Anesthesiology, Hvidovre Hospital, Copenhagen University; ^5^Section of Surgical Pathophysiology, Rigshospitalet, Copenhagen UniversityDenmark

## Abstract

**Background and purpose:**

Clinicians need valid and easily applicable predictors of outcome in patients with hip fracture. Adjusting for previously established predictors, we determined the predictive value of the New Mobility score (NMS) for in-hospital outcome in patients with hip fracture.

**Patients and methods:**

We studied 280 patients with a median age of 81 (interquartile range 72–86) years who were admitted from their own homes to a special hip fracture unit. Main outcome was the regain of independence in basic mobility, defined as. independence in getting in and out of bed, sitting down and standing up from a chair, and walking with an appropriate walking aid. The Cumulated Ambulation score was used to evaluate basic mobility. Predictor variables were NMS functional level before fracture, age, sex, fracture type, and mental and health status.

**Results:**

Except for sex, all predictor variables were statistically significant in univariate testing. In multiple logistic regression analysis, only age, NMS functional level before fracture, and fracture type were significant. Thus, patients with a low prefracture NMS and/or an intertrochanteric fracture would be 18 and 4 times more likely not to regain independence in basic mobility during the hospital stay, respectively, than patients with a high prefracture level and a cervical fracture, respectively. The model was statistically stable and correctly classified 84% of cases.

**Interpretation:**

The NMS functional level before fracture, age, and fracture type facilitate prediction of the in-hospital rehabilitation potential after hip fracture surgery.

## Introduction

The functional level before fracture appears to be the most consistent predictor of rehabilitation outcome in hip fractures in the elderly (Parker and Palmer 1993, Cree and Nade 1999, Thorngren et al. 2005). It has been stated that “the aim must be that as many patients as possible return directly home after discharge from a short hospitalization” (Thorngren et al. 1993). Within the last decade, the concept of multimodal rehabilitation (Kehlet and Wilmore 2002) has proven effective (Halbert et al. 2007) in this frail and very heterogeneous patient group. Having designated staff who optimize treatment is also effective (Parker et al. 2000, Hommel et al. 2008). Still, some patients do not regain their independence in basic mobility in the short term, which is a prerequisite for discharge directly to their own home rather than to a secondary rehabilitation facility or nursing home. Thus, a method of early and valid prediction of in-hospital rehabilitation outcome is desirable in order to adjust expectations and plan for the rehabilitation needs of each patient.

The New Mobility score (NMS) (Parker and Palmer 1993) evaluates prefracture functional level, and is a reliable (Kristensen et al. 2008) and valid predictor of long-term mortality (Parker and Palmer 1993). However, the NMS examined with a single cut-off point at 5 (range 0–9) is inferior to the Cumulated Ambulation score (Foss et al. 2006) in prediction of late rehabilitation outcome. The NMS has been used as an outcome measure (Little et al. 2008) and to compare the effect of the functional level before fracture with other variables (Foss et al. 2008).

To date, the potential of the NMS as a predictor of short-term rehabilitation outcome has not been investigated in detail. We therefore examined it as a predictor of independence in basic mobility shortly after hip fracture surgery, after adjusting for other previously established predictors before fracture and also fracture type. In addition, we tried to predict the time from surgery to independence in mobility (in days) and residential status at discharge.

## Patients and methods

### Study population

437 patients with hip fracture who were admitted consecutively to a 14-bed specialized hip fracture unit from their own home between September 2002 and July 2004 were regarded as possible participants in this prospective observational study (Figure). Inclusion in the study was restricted to patients without other fractures, who could walk independently indoors (with or without walking aids) before the fracture, who had no restrictions regarding weight bearing after surgery, and who followed the standardized program in the unit. This study was part of the Hvidovre University Hospital hip fracture project, which was approved by the Danish data protection agency.

**Figure:**
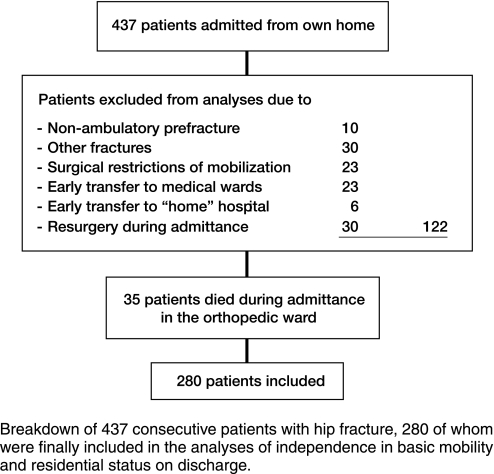
Breakdown of 437 consecutive patients with hip fracture, 280 of whom were finally included in the analyses of independence in basic mobility and residential status on discharge.

### Procedure

During their hospital stay, patients followed a well-defined care plan with multimodal fast-track rehabilitation (Foss et al. 2008) including early surgery within 24 hours of admission, epidural anesthesia and analgesia continued for 96 hours postoperatively, prophylactic intraoperative antibiotics, a standardized transfusion protocol if hemoglobin was less than 6 mmol/L, supplementary oxygen when supine in the perioperative period, low molecular weight heparin perioperatively, and enforced perioperative oral nutrition and hydration including energy and protein supplementation. Patients were mobilized on the day of surgery if possible, and an intensive physiotherapy program comprising 2 daily sessions on weekdays (once during weekends) was started on the day after surgery.

Patients were rehabilitated in the orthopedic ward, and they were discharged home when they were safely able to perform “basic mobility tasks”, defined as independence in getting in and out of bed, sitting down and standing up from a chair or toilet, and walking with the aid to be used at home. Only patients who (after intensive rehabilitation at the orthopedic ward) still required additional inpatient rehabilitation were transferred to a secondary rehabilitation facility or nursing home. Recording of the NMS by the physician is part of the normal routine in the admission ward and recording of NMS by physiotherapists is part of the routine at the stationary orthopedic ward.

### Predictor variables

6 predictor variables (age, sex, functional level before fracture, mental status, health status, and fracture type) were chosen due to their previously established influence on patient outcome after hip fracture surgery (Parker and Palmer 1995, Cree and Nade 1999, Thorngren et al. 2005, Soderqvist et al. 2006, Kristensen et al. 2009).

Prefracture functional level was evaluated with the NMS. The NMS is a composite score of the patient's ability to perform indoor walking, outdoor walking, and shopping before the hip fracture, providing a score between 0 and 3 (0 = not at all, 1 = with help from another person, 2 = with an aid, and 3 = no difficulty and no aid) for each function, resulting in a total score ranging from 0 (no walking ability at all) to 9 (fully independent) (Parker and Palmer 1993). Patients were asked about their walking ability in the last weeks before their admission with hip fracture and, if necessary, this was verified by relatives or caregivers. Previous studies have established that there is high inter-tester reliability (Kristensen et al. 2008) and determined that an NMS cutoff point of 5 is a valid predictor of 6-month functional level (Kristensen et al. 2005) and 1-year mortality (Parker and Palmer 1993). The NMS was dichotomized at all possible points of the scale to determine which dichotomization best predicted outcome variables.

Mental status was measured with a validated 9-point Danish version of the abbreviated mental test score (Qureshi and Hodkinson 1974). A score of ≤ 6 is considered to represent cognitive impairment (Parker and Palmer 1995). Accordingly, mental status was dichotomized into 2 categories, 0–6 (low) or 7–9 (high).

Health status was evaluated with the American Society of Anesthesiologists (ASA) rating (0–4) (American Society of Anesthesiologists 1963), and classified as poor (ASA 3–4) or good (ASA 1–2) (Parker and Palmer 1995).

Fracture type was classified as cervical, intertrochanteric, or subtrochanteric.

### Functional outcome variables after surgery

During the daily physiotherapy sessions, the patients were evaluated on their ambulatory capacity using the Cumulated Ambulation score (CAS), which allows day-to-day measurements of basic mobility in the early period after surgery. The CAS describes the patients' independence in 3 functions: (1) getting in and out of bed, (2) sitting down and standing up from a chair, and (3) walking ability with an appropriate walking aid. Each function is assessed on a 3-point scale (2 = independent of human assistance, 1 = requiring human assistance to perform function, 0 = unable to perform function despite human assistance). The score for each function is cumulated to provide a daily score between 0 and 6, with 6 indicating independent ambulation on that particular day. High inter-tester reliability (Kristensen et al. 2009a) and predictive validity of late rehabilitation outcome (Foss et al. 2006) for the CAS have been established in patients with hip fracture.

Whether or not the patient regained independence in basic mobility (CAS = 6 or CAS < 6), and the time from surgery to independent mobility in days (day of CAS = 6) were determined.

Residential status at discharge was classified as either the patient's own home (previous residence) or inpatient rehabilitation facilities/nursing home.

### Statistics

Continuous data on age and length of stay evaluated by the Kolmogorov-Smirnoff test were not normally distributed. Accordingly, Mann Whitney U test was used to evaluate differences between patients in regaining their independence in basic mobility or not, and discharge destination, while chi-squared test or Fischer's exact test were used for categorical data.

Simple linear regression was used to examine the influence from each predictor variable to the three outcome variables. The 6 previously established predictor variables were entered into multiple logistic regression models to determine their relative contribution to the prediction of patients not regaining independence in basic mobility or not being discharged to their previous residence. The same 6 variables were analyzed in multiple linear regression to evaluate their ability to predict the time from surgery to independent mobility in days.

Reference categories used in multiple regression analyses were: male sex, prefracture functional level (high), mental status (high), health status (ASA rating, 1–2 (healthy)), and cervical fracture, while age was entered as a continuous variable.

All analyses were done using SPSS version 16.0 and the level of significance was set at p < 0.05.

## Results

Of the 437 patients in total, 280 patients with a median age of 81 (72–86) years who followed the standardized multimodal rehabilitation program in the acute orthopedic ward were included in the analysis (Figure and Table 1). 139 (50%) had used a walking aid before fracture, and types of surgery were as follows: parallel screws or nails (n = 49), hemiarthroplasty (n = 84), dynamic hip screw (n = 129), or intramedullary hip screw (n = 18). No significant differences (p ≥ 0.1) were found for any of the predictor variables studied between patients who were included (n = 280) and those who did not follow the standardized program (n = 122) (Figure). On the other hand, the 35 patients who were not included and who died in hospital were older, had lower prefracture functional level and mental status, and a poorer health status (p ≤ 0.008) than the 280 patients who were included, while sex distribution and fracture type distribution were similar.

**Table 1.** Distribution of outcome measures according to predictor variables and length of stay (LOS), n = 280

Predictor variables	All patients n = 280	In-hospital independency in basic mobility (CAS = 6)		Discharge to own home	
		Yes n = 223 (80)	No n = 57 (20)	Yes n = 218 (78)	No n = 62 (22)
Age (years)	81 (72–86)	80 (70–85)	84 (81–91)	80 (69)	84 (81–90)
P-value		< 0.001 ^**a**^		< 0.001 ^**a**^	
Female	203 (73)	161 (79)	42 (21)	156 (77)	47 (23)
Male	77 (27)	62 (80)	15 (20)	62 (81)	15 (19)
P-value		0.8 ^**b**^		0.5 ^**b**^	
Prefracture function					
Low, NMS (0–6)	132 (47)	79 (60)	53 (40)	76 (58)	56 (42)
High, NMS (7–9)	148 (53)	144 (97)	4 (3)	142 (96)	6 (4)
P-value		< 0.001 ^**c**^		< 0.001 ^**c**^	
Low mental status	44 (16)	25 (57)	19 (43)	25 (57)	19 (43)
High mental status	236 (84)	198 (84)	38 (16)	193 (82)	43 (18)
P-value		< 0.001 ^**b**^		< 0.001 ^**b**^	
Health status					
Good (ASA score 1-2)	145 (52)	126 (87)	19 (13)	121 (83)	24 (17)
Poor (ASA score 3-4)	135 (48)	97 (72)	38 (28)	97 (72)	38 (28)
P-value		0.002 ^**b**^		0.02 ^**b**^	
Fracture type					
Cervical	148 (53)	132 (89)	16 (11)	125 (85)	23 (15)
Intertrochanteric d	132 (47)	91 (69)	41 (31)	93 (71)	39 (29)
P-value		< 0.001 ^**b**^		0.005 ^**b**^	
LOS	13 (8-21)	11 (8–16)	24 (15–33)	11 (7–16)	24 (17–36)
P-value		< 0.001 ^**a**^		< 0.001 ^**a**^	

Values are presented as median (25–75% quartiles) as number of patients (%), or as correlations.

a Mann-Whitney,

b Chi-square,

c Fischer's Exact test. d 7 patients with subtrochanteric fractures included.

CAS; Cumulated Ambulation Score, NMS; New Mobility Score,

ASA; American Society of Anaesthesiologists rating

A cutoff point at ≤ 6 for the NMS was found to be the best predictor of the 3 outcome variables. Accordingly, prefracture functional level was classified as either low (NMS ≤ 6) or high (NMS > 6) in our analyses.

Only 7 patients presented with subtrochanteric fractures and no significant differences between predictor and outcome variables were found compared to patients with intertrochanteric fractures. Accordingly, these fracture types were pooled in the analyses.

### Functional outcome after surgery

223 patients (80%) achieved independence in basic mobility during their hospital stay, with 208 (93%) regaining this independence within 2 weeks of surgery. Except for sex, all predictor variables were associated (p ≤ 0.002) with regain of independence in basic mobility in univariate testing (Table 1). When all 6 variables were entered into a multiple logistic regression analysis, only age, low prefracture functional level (NMS ≤ 6), and having an intertrochanteric fracture remained significant predictors of patients not regaining independence in basic mobility (Table 2). The model developed from this analysis correctly predicted 84% of cases, and further examination of 4 cases with residuals above 2 showed no influence on the model. Otherwise, residuals were normally distributed.

**Table 2.** Simple and multiple logistic regression analysis of patients with hip fracture not regaining independency in basic mobility (CAS < 6), during admittance, n = 280

Predictor variables	Crude OR		Adjusted OR	
	(95% CI)	P-value	(95% CI)	P-value
Age (continuous)	1.090 (1.048–1.132)	< 0.001	1.046 (1.001–1.093)	0.04
Female sex	1 (0.6–2.1)	0.8	0.2 (0.2–1.3)	0.2
Low prefracture functional level (NMS 0–6)	24 (8–69)	< 0.001	18 (6–55)	< 0.001
Low mental status	4.0 (2–8)	< 0.001	2.0 (0.8–4.5)	0.1
Poor health status (ASA 3–4)	2.0 (1.1–3.6)	0.02	1.5 (0.7–3.3)	0.3
Intertrochanteric fracture	2.3 (1.3–4.1)	0.005	4.2 (2–9)	< 0.001

OR; odds ratio, CI; confidence interval. Other abbreviations presented in Table 1.

### Time from surgery to independence in mobility

Of the 5 variables that could significantly predict the number of days to independence in mobility in univariate testing (Table 3), older age, a low prefracture functional level, and having an intertrochanteric fracture were significantly associated with regaining independence in mobility in the later postoperative period in multivariate linear regression analysis. It was of some concern that 9 cases had residuals above 3 and the model only explained 26% of the variance in the number of days to independence in mobility. Accordingly, a new regression analysis was performed without these 9 cases. The 3 same variables remained significant (p < 0.001) in the new model (Table 3) with no outliers or multicollinearity, and the R2 value showed that the new model could explain 42% of the variance in days from surgery to independence in mobility. Also, residuals were normally distributed in the latter model.

**Table 3.** Simple and multiple linear regression analysis of time from surgery to independent mobility in days (CAS = 6) in the orthopedic ward, n = 214

Predictor variables	Crude B-values		Adjusted B-values	
	(95% CI)	P-value	(95% CI)	P-value
Age (continuous)	0.1 (0.1–0.2)	< 0.001	0.1 (0.06–0.13)	< 0.001
Female sex	1.5 (0.5–2.6)	0.005	0.03 (–0.9–1.0)	1
Low prefracture functional				
Level (NMS 0–6)	3.4 (2.5–4.3)	< 0.001	2.7 (1.8–3.6)	< 0.001
Low mental status	1.0 (-0.5–2.6)	0.2	0.3 (–0.9–1.5)	0.6
Poor health status (ASA 3–4)	1.0 (1.2–3.1)	0.04	0.3 (–0.5–1.1)	0.5
Intertrochanteric fracture	2.1 (1.2–3.1)	< 0.001	2.3 (1.5–3.1)	< 0.001
Constant	–	–	-9.1 (–12.2–6)	< 0.001

CI; confidence interval. Other abbreviations presented in Table 1.

### Residential status on discharge

218 patients (78%) were discharged to their own homes, while 47 (17%) were discharged to further inpatient rehabilitation and 15 (5%) were sent to a nursing home (permanently). Predictor variables for which there was a statistically significant association with discharge destination in univariate testing were: age, prefracture functional level, mental status, health status, and fracture type (Table 1). When these variables and sex were entered into multiple logistic regression, only older age (p = 0.01), having a low prefracture functional level (p < 0.001), and intertrochanteric fracture (p = 0.02) remained significant predictors of patients not being discharged to their own home (Table 4). 5 cases with residuals above 2 did not influence the model. Otherwise, residuals were normally distributed, and the model correctly classified 81% of cases.

**Table 4.** Simple and multiple logistic regression analysis of patients not being discharged directly to their own home from the orthopedic ward, n = 280

Predictor variables	Crude OR		Adjusted OR	
	(95% CI)	P-value	(95% CI)	P-value
Age (continuous)	1.097 (1.055–1.140)	< 0.001	1.056 (1.013–1.102)	0.010
Female sex	1.2 (0.6–2.4)	0.5	0.5 (0.2–1.3)	0.2
Low prefracture functional				
Level (NMS 0–6)	18 (7–43)	< 0.001	13 (5–33)	< 0.001
Low mental status	3.3 (1.7–6.7)	< 0.001	1.6 (0.7–3.6)	0.3
Poor health status (ASA 3–4)	2.0 (1.1–3.6)	0.02	1.1 (0.5–2.2)	0.9
Intertrochanteric fracture	2.3 (1.3–4.1)	0.005	2.2 (1.1–4.4)	0.02

OR; odds ratio, CI; confidence interval. Other abbreviations presented in Table 1

Additional analyses of the association between prefracture functional level and fracture type on the one hand and other predictor variables on the other showed that patients with an intertrochanteric fracture were generally older than patients with a cervical fracture (p = 0.03). No other significant differences were found according to fracture type. Patients with a low prefracture functional level were more often women, were generally older, had a lower mental status, and a poorer health status than patients with a high functional level before fracture.

## Discussion

We found that the functional level before fracture, assessed by the NMS, is a strong and independent predictor of in-hospital outcome in patients with hip fracture, when adjusted for previously established predictors. Furthermore, fracture type and age were the only other predictor variables that were independently associated with short-term outcome in multiple regression analysis.

### Functional level before fracture

A patient with a low prefracture functional level (NMS ≤ 6) was 18 times more likely not to regain independence in basic mobility during hospitalization, regained independence in mobility (if at all) on average 3 days later, and was 13 times more likely not to be discharged directly to his or her own home, compared to a patient with a high functional level before fracture (NMS > 6). The importance of the prefracture functional level in our study confirms previous findings (Parker and Palmer 1995, Cree and Nade 1999, Cornwall et al. 2004, Thorngren et al. 2005). Most of these studies evaluated discharge status or long-term outcome, and not the re-establishment of their independence in basic mobility while still in hospital. Also, rather than using the NMS, the prefracture functional level in previous studies was evaluated with more complex scores such as the Katz score (Soderqvist et al. 2006), the Functional Independency measure (Cornwall et al. 2004), and the Functional Recovery score (Zuckerman et al. 2000). Another study presented a high classification accuracy in a multiple logistic regression model with 50 patients, predicting independency in transfers and ambulation within 2 weeks of their hip fracture (Duke and Keating 2002).

### Fracture type

Having an intertrochanteric fracture rather than a cervical one was an independent predictor of our 3 outcome variables in multiple regression analysis (Tables 2, 3, and 4). Thus, a patient with an intertrochanteric fracture was 4 times more likely not to regain independence in basic mobility, regained it on average 2 days later, and was twice as likely not to be discharged to his or her own home, compared to a patient with a cervical fracture. This influence of fracture type on outcome and early discharge destination is in accordance with previous studies (Thorngren et al. 1993, Koval et al. 1995, Kristensen et al. 2009c). We have found no other multiple regression models with reports of increased odds of not regaining independence in basic mobility from having an intertrochanteric fracture, as compared to having a cervical fracture. A possible explanation of this finding may be larger edema in the thigh with an intertrochanteric fracture, with pain and reduced knee-extension strength (Foss et al. 2009, Kristensen et al. 2009b).

### Age

Older age was independently associated with not regaining independence in basic mobility, a greater number of postoperative days to independence in mobility, and not being discharged to one's own home. Thus, an 80-year-old patient compared to one at 70 has a 50% and 60% increase in odds, respectively, of not regaining independence in basic mobility and being discharged directly to his or her own home. Our finding that age is important confirms the results of previous studies (Parker and Palmer 1995, Cree and Nade 1999, Thorngren et al. 2005). The 3 other predictor variables: sex, mental status, and health status did not reach statistical significance in any of the multiple regression models.

Our findings indicate that the factors age, prefracture functional level (easily assessed using the NMS before or just after surgery), and fracture type are sufficient for prediction of the short-term outcome for most patients. Thus, a good estimate of in-hospital outcome is possible from the very first contact with the patient. In patients who are especially at risk of not regaining independence in basic mobility, special attention and a more intensive training program may improve outcome. Also, our findings may be used to stratify patients who are entered into research studies of short-term outcome.

Previous studies to evaluate prefracture predictors of short-term outcome in patients with hip fracture have been conducted in settings different from our well-defined setup (Foss et al. 2008), and often with selected groups of patients, which makes between-study comparisons difficult. Our prospective study included data on all 437 patients admitted to the unit from their own home, although detailed analyses were only made for those patients who followed the standardized program (n = 280). The 3 regression models used were statistically stable and the sample size was large. However, the clinical use of these models in other settings will require confirmation from other prospective studies, some of which should also examine the influence of postoperative events.
